# Warburg effect enhanced by AKR1B10 promotes acquired resistance to pemetrexed in lung cancer-derived brain metastasis

**DOI:** 10.1186/s12967-023-04403-0

**Published:** 2023-08-16

**Authors:** Wenzhe Duan, Wenwen Liu, Shengkai Xia, Yang Zhou, Mengyi Tang, Mingxin Xu, Manqing Lin, Xinyu Li, Qi Wang

**Affiliations:** 1 Department of Respiratory Medicine, The Second Hospital, Dalian Medical University, Dalian, China; 2 Cancer Translational Medicine Research Center, The Second Hospital, Dalian Medical University, Dalian, China; 3 Ningbo Institute of Innovation for Combined Medicine and Engineering, The Affiliated Li Huili Hospital, Ningbo University, Ningbo, China; 4 Department of Neurosurgery, The Second Hospital, Dalian Medical University, Dalian, China

**Keywords:** Lung cancer, Brain metastasis, Chemotherapeutic resistance, Pemetrexed, AKR1B10, Warburg metabolism, Histone lactylation

## Abstract

**Background:**

Resistance to pemetrexed (PEM), a rare chemotherapeutic agent that can efficiently cross the blood-brain barrier, limits the therapeutic efficacy for patients with lung cancer brain metastasis (BM). Aldo-keto reductase family 1 B10 (AKR1B10) was recently found to be elevated in lung cancer BM. The link between AKR1B10 and BM-acquired PEM is unknown.

**Methods:**

PEM drug-sensitivity was assessed in the preclinical BM model of PC9 lung adenocarcinoma cells and the BM cells with or without AKR1B10 interference in vitro and in vivo*.* Metabolic reprogramming of BM attributed to AKR1B10 was identified by chromatography-mass spectrometry (GC-MS) metabolomics, and the mechanism of how AKR1B10 mediates PEM chemoresistance via a way of modified metabolism was revealed by RNA sequencing as well as further molecular biology experimental approaches.

**Results:**

The lung cancer brain metastatic subpopulation cells (PC9-BrM3) exhibited significant resistance to PEM and silencing AKR1B10 in PC9-BrM3 increased the PEM sensitivity in vitro and in vivo. Metabolic profiling revealed that AKR1B10 prominently facilitated the Warburg metabolism characterized by the overproduction of lactate. Glycolysis regulated by AKR1B10 is vital for the resistance to PEM. In mechanism, AKR1B10 promoted glycolysis by regulating the expression of lactate dehydrogenase (LDHA) and the increased lactate, acts as a precursor that stimulates histone lactylation (H4K12la), activated the transcription of *CCNB1* and accelerated the DNA replication and cell cycle.

**Conclusions:**

Our finding demonstrates that AKR1B10/glycolysis/H4K12la/*CCNB1* promotes acquired PEM chemoresistance in lung cancer BM, providing novel strategies to sensitize PEM response in the treatment of lung cancer patients suffering from BM.

**Supplementary Information:**

The online version contains supplementary material available at 10.1186/s12967-023-04403-0.

## Introduction

Brain metastasis (BM) is a malignant event leading to poor prognosis in non-small cell lung cancer (NSCLC) patients; the limited treatment modalities and poor treatment outcomes are the main factors contributing to its poor prognosis (median survival: 4–6 months) [1, 2]. The current common treatment strategy for BM is a combination of applicable options including surgery, radiotherapy, chemotherapy, molecular targeted therapy and anti-angiogenic therapy [3]. Pemetrexed (PEM)-platinum, remains the first-line chemotherapy in patients with metastatic NSCLC [4]. Particularly, PEM has shown a greater ability to penetrate the blood-brain barrier (BBB) among chemotherapy agents, indicating a powerful potential in the treatment of BM. However, the response rate is not as effective as expected in BM populations [5–7]. Therefore, it’s important to investigate the intrinsic mechanisms that contribute to the unfavorable response to PEM in metastatic tumor cells.

Aldo-keto reductase family 1 B10 (AKR1B10) is a member of the aldo-keto reductase superfamily. Under physiological conditions, it is mainly expressed in the intestinal system and exerts a cyto-detoxification effect as a reductase to protect cells from carbonyl damage [8]. In the last decade, the prominent role of AKR1B10 in malignancies has received widespread attention [9]. Increasing evidences have indicated that AKR1B10 takes part in the acquirement of chemo-resistance in several cancers, including hepatocellular carcinoma(HCC) [10–12], gastrointestinal cancer [13] and lung cancer [12, 14]. Noteworthy, AKR1B10 has been demonstrated to be significantly overexpressed in a lung cancer cell line with high BM potential and NSCLC patients with BM in our previous work [15]. However, whether there is any association between elevated AKR1B10 expression and poor PEM drug response in lung cancer BM is unknown.

In this study, we found that brain metastatic lung cancer cells develop significant resistance to PEM and AKR1B10 plays a vital role in this acquirement of PEM resistance. By conducting concomitant metabolomics and RNA-sequencing study in BM cells with or without AKR1B10 genetic silence, we further revealed the underlying mechanism that AKR1B10 facilitates the Warburg metabolism by regulating the transcription of LDHA, and the resulting increase in lactate induces the transcriptional activation of the cell-cycle related gene *CCNB1* through lactate-dependent histone modification, leading to the acquired PEM resistance.

## Materials and methods

### Cells and reagents

The human lung cancer cell line PC9 was purchased from the Chinese Academy of Medical Sciences (Beijing, China). The highly brain metastatic derivative (PC9-BrM3) was constructed by planting the parental cells PC9 into immunodeficient mice in a way of left ventricular injection and metastatic cells were extracted from harvested brain metastases; these injection-isolation-expansion cycling operations were repeated three times as previously indicated [15]. All cells were routinely cultured in Roswell Park Memorial Institute medium-1640 (RPMI1640) supplemented with 10% fetal bovine serum (FBS), 100 U/mL penicillin and streptomycin (all these reagents were obtained from Gibco, Invitrogen, Inc, California, USA) and maintained in a humidified atmosphere with 5% CO2 at 37 °C. Pemetrexed Disodium (MB1183), 2-Deoxy-d-glucose (2-DG, MB5453) were purchased from Meilunbio (Dalian, China), Sodium lactate (L7022) was purchased from MERCK (Germany).

### Cell viability and colony formation assay

Cell viability was measured by Cell Counting Kit-8 (K1018, ApexBio, Houston, USA) following the manufacturer’s instructions. For colony formation, cells were harvested and seeded in 6-well plates at a density of 1000 cells per well. After 10-days culture, cell colonies were fixed with 4% paraformaldehyde (P1110, Solarbio, Beijing, China) for 10 min and stained with 1% crystal violet (G1062, Solarbio, Beijing, China) for 20 min.

### Analysis of cell apoptosis

Cell apoptosis was assessed by flow cytometry. Briefly, cells were labeled by Annexin V-APC and 7-AAD dual staining according to the manufacturer’s instructions (E-CK-A218, Elabscience, Wuhan, China). Subsequently labeled cells were collected and analyzed using a flow cytometer (AccuriC6, BD Biosciences).

### RNA interference (RNAi) and plasmid design and transfection

The AKR1B10-targeting shRNA and its negative control shRNA (shNC) constructed in the LV2 lentiviral vector, and lentiviral particles expressing luciferase infused shRNA were packaged in 293T cells (GenePharma, Suzhou, China). The sequences of shRNAs were 5’-CACGCATTGTTGAGAACAT-3’(sh1) and 5’-GTGCCTATGTCTATCAGAA-3’ (sh2). The target sequences of AKR1B10 plasmid were referred to its genetic sequence in the PubMed (https://www.ncbi.nlm.nih.gov/gene). Transfection was carried out as directed by the manufacturer. The knockdown or overexpression efficiency was validated by western blot analysis.

### Western blot analysis

Cell proteins were extracted by RIPA lysis buffer (Meilunbio, Dalian, China) containing a protease inhibitor cocktail and a phosphatase inhibitor cocktail (Sigma, USA). The concentration of protein was assessed using the BCA assay kit (Thermo Fisher Scientific Inc., USA). Protein lysates were then separated by sodium dodecyl sulfate-polyacrylamide gel electrophoresis (SDS-PAGE) and transferred onto nitrocellulose membranes (Millipore, Billerica, USA). The membranes were blocked and then incubated with primary antibodies against AKR1B10 (1:2000; ab192865, Abcam, UK), LDHA(1:2000, ab53488, Abcam, UK), PI3K (1:1000 dilution; #4257, Cell Signaling Technology, USA), phospho-PI3K (1:1000 dilution; #17,366, Cell Signaling Technology, USA), AKT (1:1000 dilution; #13,038, Cell Signaling Technology, USA), phospho-AKT (1:1000 dilution; #4060, Cell Signaling Technology, USA), histone lactylation (Lactyl-Histone Antibody Sampler Kit, 1:2000, PTM-7093, PTM Bio, China) and β-actin (1:10,000; 66009-1-Ig, Proteintech, USA). After washing with 0.05% Tris-buffered saline/Tween-20 (TBST), the corresponding secondary antibodies conjugated with horseradish peroxidase (1:5000; Proteintech, USA) were further used. The ECL western blotting substrate (Tanon, China) was used to analyze the chemiluminescence of the blots. Protein expression was quantified by Image J software (National Institutes of Health, USA).

### Animal studies

This work was approved by the Animal Ethics Review Committee of Dalian Medical University (No.00122773). Female BALB-c-nu mice (4–6 weeks) were purchased from the Beijing Vital River Laboratory Animal Technology Co. Ltd., China. After anesthetizing with 2.5% Tribromoethanol (5 mg/kg body weight; Sigma, Missouri, USA), 100,000 PC9-BrM3 cells with or without AKR1B10 knockdown (shAKR1B10 or shNC) in 3 µL PBS were injected into the cerebrum of each mouse by using a stereotactic machine as previously described [16]. Brain colonization was monitored in vivo by bioluminescence imaging (BLI) weekly. Briefly, mice were anesthetized and injected intraperitoneally with D-Luciferin (150 mg/kg body weight; Promega, Wisconsin, USA), images were acquired using an IVIS Spectrum Xenogen machine (PerkinElmer, Massachusetts, USA). The Living Image software (version 2.50) was used to analyze the bioluminescence images. Pemetrexed disodium administration (n = 3, 100 mg/kg, once every 5 days, intraperitoneal injection) was initiated after confirmation of colonization and the brain metastases was monitored in vivo by BLI weekly for 4 weeks.

### Metabolomics analysis

GC-MS-based cell metabolic profiling was performed similarly as previously described [17, 18]. In brief, cells were harvested with 1 mL of methanol/water solution (4:1, v/v) and the lysates was subsequently lyophilized. The lyophilized samples were treated with oximation and silylation reactions before GC-MS analysis. Quality-control (QC) samples were prepared and pretreated as the samples. GC-MS analysis was performed using a GCMS-QP2010 plus system (Shimadzu, Kyoto, Japan) coupled with a DB-5 MS fused-silica capillary column (30 m × 0.25 mm × 0.25 μm, Agilent Technologies, Palo Alto, CA). 1 µL of samples was injected with a split ratio of 1:20. The linear velocity of carrier gas (Helium, 99.9995%, China) was set at 40 cm/s. The oven temperature was kept at 80 °C for 1 min and raised to 210 °C at increment of 30 °C/min, then to 320 °C at increment of 20 °C/min and kept for 4 min. An electron ionization source (EI, 70 eV) was used for ionization. The data acquisition started at 2.92 min with the mass scan range of 50–600 m/z and the event time of 0.2 s. The temperatures of the inlet, the transfer line and the ion source were 320, 300 and 230 °C, respectively. ChromTOF 4.43 (LECO, Saint Joseph, USA) and GC−MS browser (Shimadzu, Kyoto, Japan) software were used to identify and quantify the metabolic features. The intensity of metabolic features was normalized to the total peak areas of raw data from cell samples. Five-six replicates were set for each group as biological replicates.

### Measurement of pyruvate and lactate

Pyruvic Acid Colorimetric Assay Kit (E-BC-K130-M, Elabscience, Wuhan, China) and L-Lactic Acid Colorimetric Assay Kit (E-BC-K044-M, Elabscience, Wuhan, China) were used to assess the level of pyruvate and lactate in cell lysates as directed by manufacturer’s instructions. Data were standardized by the corresponding protein concentration which was determined by BCA assay kit.

### RNA-sequencing analysis

This work was supported by the Novogene (Beijing, China) Co. Ltd. The main experimental procedures of RNA-sequencing analysis, which include RNA quantification and qualification, library preparation for transcriptome sequencing trypsin digestion, clustering and sequencing and data analysis, are presented in the Supplementary methods in detail.

### Quantitative-polymerase chain reaction (q-PCR)

Total RNA was extracted using the Trizol® reagent (Transgen biotech, China) and quantitated at OD_260_nm. Total RNA (1.0 µg) was treated with RNase-free DNase I and reverse-transcribed into cDNA using random primers and Superscript II® retrotranscriptase (Transgen biotech, China). The resulting cDNAs were mixed with the SYBR PCR master mix (Transgen biotech, China) and run on the StepOnePlus Applied Biosystems Realtime PCR machine (Roche, Swiss). One cycle of a denaturing step (3 min at 95 °C) was applied, which was followed by 40 cycles of amplification (12 s at 95 °C, 30s at 62 °C and 30s at 72 °C), with fluorescence measured during the extension. Primers used in this study are as follows:

**GAPDH** 5′-CATGAGAAGTATGACAACAGCCT-3′(forward);

5′-AGTCCTTCCACGATACCAAAGT-3′(reverse);

**LDHA** 5′-AAGCGGTTGCAATCTGGATT-3′(forward);

5′-GAGACACCAGCAACATTCATTCC-3′(reverse);

**LDHB**5′-GCTGCCATGGATGGATTTTG-3′(forward);

5′-CCATCTTATGCACTTCCTTCCAA-3′(reverse);

**CCNB1** 5′-AAGAGCAAGCAGTCAGACCA-3′(forward).

5′-TTCTTAGCCAGGTGCTGCATA-3′(reverse);

**CDK1** 5′-CTGGGGTCAGCTCGTTACTC-3′(forward).

5′-TCCACTTCTGGCCACACTTC-3′(reverse);

**RAD21** 5′-TGCTTCATGGTCTTCAGCGT-3′(forward);

5′-GCTGTAGAACTTTGCGGCAG-3′(reverse).

The relative quantification value of mRNA expression was calculated using the comparative CT (^ΔΔ^CT) method and Step-OnePlus software v2.0.1 (Applied Biosystems, USA) with *GAPDH* as an internal control.

### Clinical tissue specimens

Tissue specimens were harvested from the Second Hospital of Dalian Medical University. Written informed consent was obtained from all participants. This study was approved by the Ethics Review Committee of the Second Hospital of Dalian Medical University.

### Immunohistochemistry (lHC)

 Briefly, paraffin tissue sections (3 μm) were dewaxed, hydrated, and incubated with 3% H_2_0_2_ in methanol and subjected to antigen retrieval by TE buffer or sodium citrate. The sections were blocked with 5% goat serum, probed overnight with primary antibodies for anti-AKR1B10 antibody (1:500, Abcam, ab192865) and anti-LDHA antibody (1:2000, Abcam, ab52488). Tissue sections were reacted with biotinylated secondary antibodies and detected by the Streptavidin. Peroxidase lHC assay kit and diaminobenzidine (DAB). Two independent pathologists evaluated the immunostaining in a blinded fashion and performed the scoring. Immunohistochemical staining was quantified by the immunoreactive scores (IRS) of Remmele and Stegner [19].

### Analysis of cell cycle

Cell cycle was assessed by flow cytometry. Briefly, cells were labeled by propidium (PI) staining according to the manufacturer’s instructions (C1052, Beyotime, China). Subsequently labeled cells were collected and analyzed using a Flow Cytometer (AccuriC6, BD Biosciences).

### Histone extraction

Histone was extracted by using histone extraction kit (ab113476, Abcam, UK) according to the manufacturer’s instructions.

### Chromatin immunoprecipitation (ChIP)

ChIP analysis was performed using the ChIP kit (#53,040, Active-Motif, USA) as per the manufacturing guidelines. Chromatin extracts containing DNA fragments were immunoprecipitated with 6 ug of monoclonal anti-L-Lactyl-Histone H4 (Lys12) Rabbit mAb antibody (PTM-1411RM, PTM bio, China) or normal IgG (as a control). The primers used for the qPCR analysis in this study are listed in Additional file 1: Table S1.

### Statistical analysis

For cell metabolomics, the unsupervised principal component analysis (PCA) model with unit variance (UV) scaling was analyzed by SIMCA 14.1 (Umetrics, Umea, Sweden) to explore metabolic differences of inter-group. Univariate analysis was performed by the Mann–Whitney U test and false discovery rate (FDR) correction [20] using the MeV software package (version 4.8.1). The metabolites with *p-*value < 0.05 and FDR < 0.15 were defined as significantly changed and displayed in the heat map using the MeV software package (version 4.8.1). On the basis of the significantly differential metabolites, pathway analysis was operated on MetaboAnalyst website (http://www.metaboanalyst.ca) to find the significantly differential pathways [21].

For RNA-sequencing, differential expression analysis was performed using the DESeq2 R package (1.20.0) where genes with an adjusted p-value < 0.05 found by DESeq2 were assigned as differentially expressed, and ClusterProfiler R package was used for Gene Ontology (GO) and KEGG enrichment analysis [22, 23]. The codes reference resource was provided in the Additional file 2: Table S2.

GraphPad Prism software 8.0 was used for statistical analysis. Quantitative data are presented as mean (± standard deviation) values from at least 3 independent experiments. Differences between two groups were assessed using the *t*-test. The correlation between AKR1B10 and LDHA staining scores in tissue samples was assessed using the Spearman’s rank correlation test.

## Results

### Highly brain metastatic lung cancer cells achieved prominent resistance to PEM

**Fig. 1:**
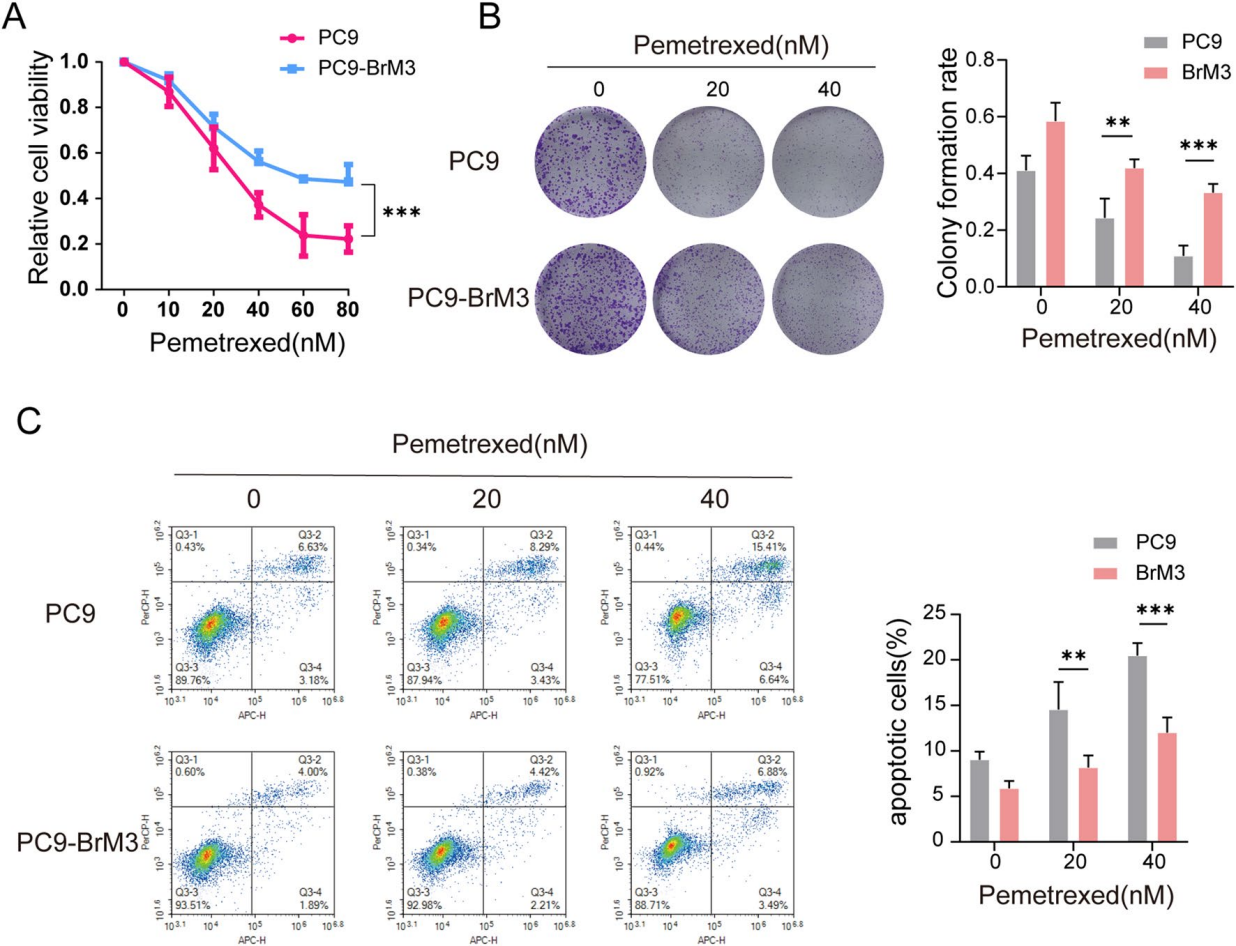
Highly brain metastatic lung cancer cells achieved prominent resistance to PEM. **A** PC9 and PC9-BrM3 cells were treated with different doses of PEM for 72 h and CCK-8 assays were performed to determine their viability. **B** Survival of PC9 and PC9-BrM3 cells after treatment with certain concentrations of PEM (0, 20, 40 nM) and the results of clonogenic assays showing the treatment effect. Pixel density quantification of clonogenic assays is shown as histogram. **C** Flow cytometry analysis of PC9 and PC9-BrM3 cells treated with PEM (20 nM) for 24 h. (n = 3, **p < 0.01, ***p < 0.001)

The highly brain metastatic lung cancer cells PC9-BrM3, which were derived from the parental PC9 cells and exhibited high propensity for BM, were established by us in previous work [15]. We evaluated the PEM drug sensitivity in PC9 and its highly brain metastatic subpopulation PC9-BrM3 by treating these two groups of cells with pemetrexed disodium. The results of cell viability assay and colony formation assay indicated significant PEM resistance of the highly brain metastatic PC9-BrM3 compared with the parental PC9 group (Fig. 1A, B). Annexin-V assay showed that the apoptotic cells induced by PEM were significantly decreased in PC9-BrM3 (Fig. 1C), revealing similar trends to those observed in the cell viability and colony formation assays. Together, the results indicated significant PEM chemotherapeutic resistance of the brain metastatic lung cancer cells, suggesting that the intrinsic acquired alterations in brain metastatic cells are the main cause of poor chemotherapy outcomes.

### Suppression of AKR1B10 attenuated PEM-chemoresistance of BM cells in vitro and in vivo

**Fig. 2:**
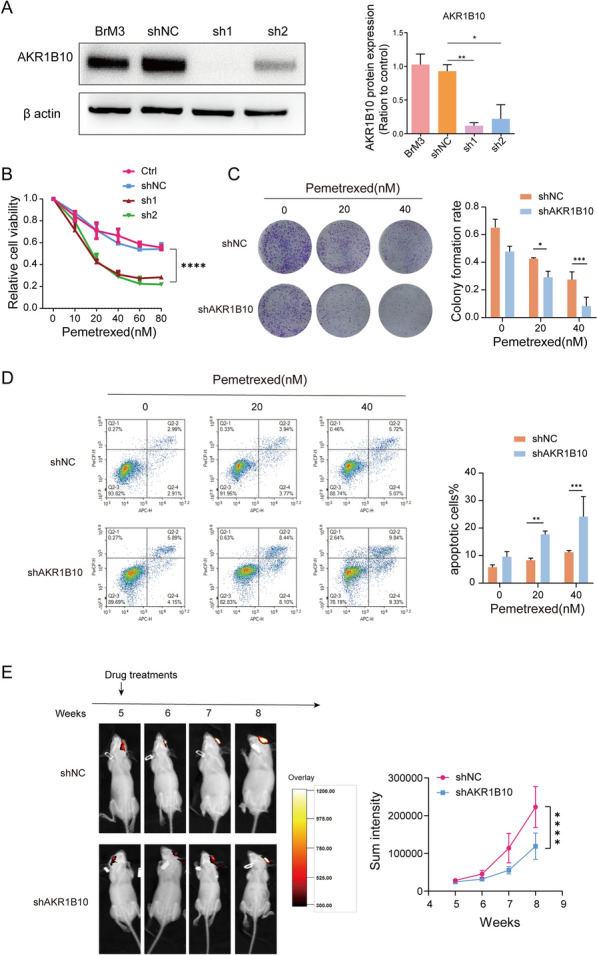
Suppression of AKR1B10 attenuated PEM-chemoresistance of BM cells in vitro and in vivo. **A** Western blotting showing the AKR1B10 knockdown efficiencies in PC9-BrM3 cells. **B** Indicated AKR1B10 knockdown PC9-BrM3 cells were treated with different doses of PEM for 72 h and CCK-8 assays were performed to determine their viability. **C** Results of clonogenic assays showing cell survival of the AKR1B10 knockdown PC9-BrM3 cells after treatment with certain concentrations of PEM (0, 20, 40 nM). Pixel density quantification of clonogenic assays is shown as histogram. **D** Flow cytometry analysis of AKR1B10 knockdown PC9-BrM3 cells treated with PEM (20 nM) for 24 h. **E** Heat map image representations of bioluminescence intensity for representative mice from the indicated groups of the therapy response experiment. Nude mice were implanted with PC9-BrM3 cells with or without AKR1B10 knockdown (10^5^ cells/mouse) by intracranial injection. PEM Treatments (n = 3, 100 mg/kg, once a week, i.p.) were initiated at the 3th week. Bioluminescence intensity in the same bioluminescence heat map range was measured every week. Plot of mean bioluminescence readings for control and treatment group mice; the standard error is indicated for each imaging point. (n = 3, **p < 0.01, **p < 0.0001, Ctrl, control PC9-BrM3 cells; shNC, PC9-BrM3 cells transfected with negative control shRNA; sh1, PC9-BrM3 cells transfected with AKR1B10-targeted shRNA vector 1; sh2, PC9-BrM3 cells transfected with AKR1B10-targeted shRNA vector 2; shAKR1B10, PC9-BrM3 cells transfected with AKR1B10-targeted shRNA vector 1)

Since our previous work had found that AKR1B10 was significantly elevated either in PC9-BrM3 cells or in tissue and serum samples of NSCLC patients with BM, we verified the potential association between up-regulated AKR1B10 protein expression and the observed acquired drug resistance of brain metastatic cells. AKR1B10 was silenced with two different shRNAs (sh1 and sh2) in PC9-BrM3 cells (Fig. 2A) and the PEM sensitivities were then evaluated in vitro and *in vivo.*

The results of cell viability assays, clonogenicity assays and cell apoptosis assays were similar and all indicated that suppression of AKR1B10 significantly enhanced the response of PC9-BrM3 to PEM (Fig. 2B–D). We further confirmed the effect of AKR1B10 on the PEM therapeutic response in vivo. PC9-BrM3 cells with or without AKR1B10 knockdown (BrM3-shAKR1B10 or BrM3-shNC) were implanted into nude mice by intracranial injection respectively. Once the tumor mass was confirmed, mice were administered with PEM weekly. The regular BLI analysis showed a consistent result with in vitro assays that BrM3-shAKR1B10 was remarkably sensitive to PEM than BrM3-shNC cells in vivo (Fig. 2E). Collectively, these data indicated that inhibition of AKR1B10 rendered the brain metastatic cells more responsive to PEM.

### Metabolic profiling revealed that AKR1B10 significantly augmented the Warburg effect

**Fig. 3:**
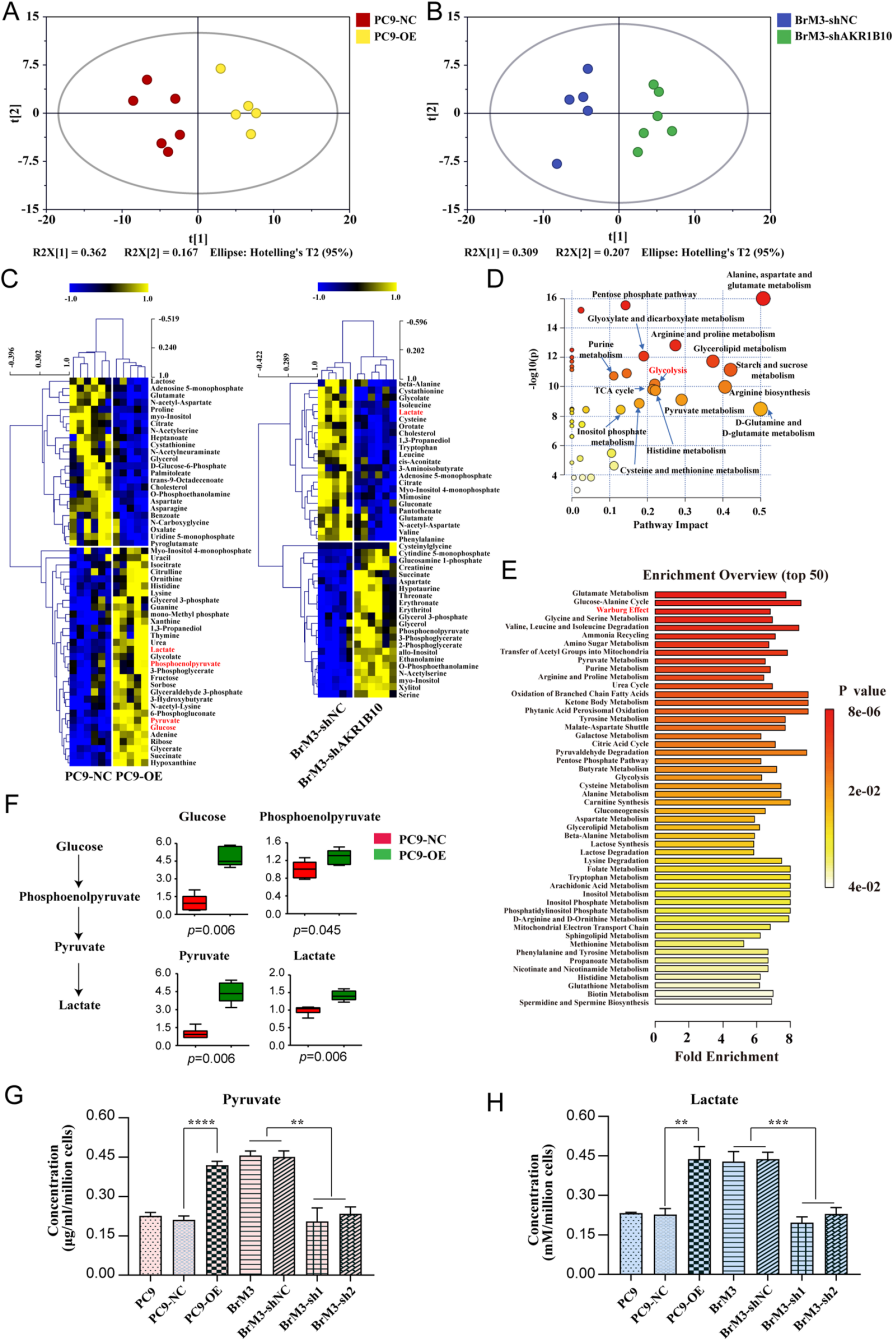
Metabolic profiling revealed that AKR1B10 significantly augmented the Warburg effect. **A** The PCA score scatter plot with UV scaling of PC9-OE and PC9-NC cells. R2X = 0.529, Q2 = 0.208. **B** The PCA score scatter plot with UV scaling of BrM3-shAKR1B10 and BrM3-shNC cells. R2X = 0.515, Q2 = 0.0448. **C** Heat map of focused differential annotated metabolites either in PC9-OE cells compared with PC9-NC cells, or in BrM3- shAKR1B10 cells compared with BrM3-shNC cells. **D**, **E** The differential metabolic pathway (**D**) and enriched KEGG pathways (**E**) in PC9-OE cells compared with PC9-NC cells. **F** The fold changes of glucose, phosphoenolpyruvate, pyruvate and lactate in PC9-OE cells compared with PC9-NC cells. **G**, **H** Cellular concentrations of pyruvate (**G**) and lactate (**H**) in indicted groups. (n = 3, **p < 0.01, ***p < 0.001, ****p < 0.0001, PC9-NC, PC9 transfected with negative control plasmid; PC9-OE, PC9 transfected with AKR1B10 plasmid. shNC, PC9-BrM3 cells transfected with negative control shRNA; sh1, PC9-BrM3 cells transfected with AKR1B10-targeted shRNA vector 1; sh2, PC9-BrM3 cells transfected with AKR1B10-targeted shRNA vector 2; shAKR1B10, PC9-BrM3 cells transfected with AKR1B10-targeted shRNA vector 1)

Increasing studies have given solid evidence that enzymatic protein alter organism phenotype by reprogramming cellular metabolism [24, 25]. To explore the endogenous metabolic changes brought by AKR1B10 in brain metastatic cells, we performed metabolomics in PC9-BrM3 cells with or without AKR1B10 silence (BrM3-shNC/shAKR1B10), and parental PC9 cells with or without AKR1B10 overexpression and its parental cells (PC9-NC/OE). A typical total ion chromatogram of cell metabolic profile was presented (Additional file 5: Fig. 1A). And 83.2 and 91.3% of features in QC samples had the relative standard deviation (RSD) distributions less than 20 and 30%, respectively (Additional file 5: Fig. 1B), demonstrating a good data quality of the work. The unsupervised PCA model displayed significantly different global changes in metabolic profile in each cell group (Fig. 3A, B), indicating a metabolic shifting caused by AKR1B10. Univariate analysis revealed different metabolites either in PC9-OE cells compared with PC9-NC cells, or in BrM3- shAKR1B10 cells compared with BrM3-shNC cells (Fig. 3C, Additional file 3: Table S3). We found that the levels of lactate, the end product of glycolysis, were accompanied by changes in AKR1B10. To reveal the metabolic disturbance by AKR1B10, we analyzed the differential metabolic pathway and listed the enriched KEGG pathways by mapping the different metabolites between PC9-OE and PC9-NC cell groups (Fig. 3D, E). The results indicated that AKR1B10 significantly influenced the Warburg effect (aerobic glycolysis) in tumor cells. To visualize the influence on the Warburg effect caused by AKR1B10, the changes of differential products (glucose, phosphoenolpyruvate, pyruvate, lactate) involved in the glycolytic pathway were displayed respectively (Fig. 3F), indicating a hyperactive Warburg effect in lung cancer cells with AKR1B10 overexpressed. We further verified the intracellular levels of pyruvate and lactate by kits and the results were consistent with the metabolomics (Fig. 3G, H). In general, these data suggested that AKR1B10 remarkably facilitates the Warburg effect in lung cancer BM cells.

#### AKR1B10-mediated PEM resistance is dependent on the enhanced effects on anaerobic glycolysis

**Fig. 4:**
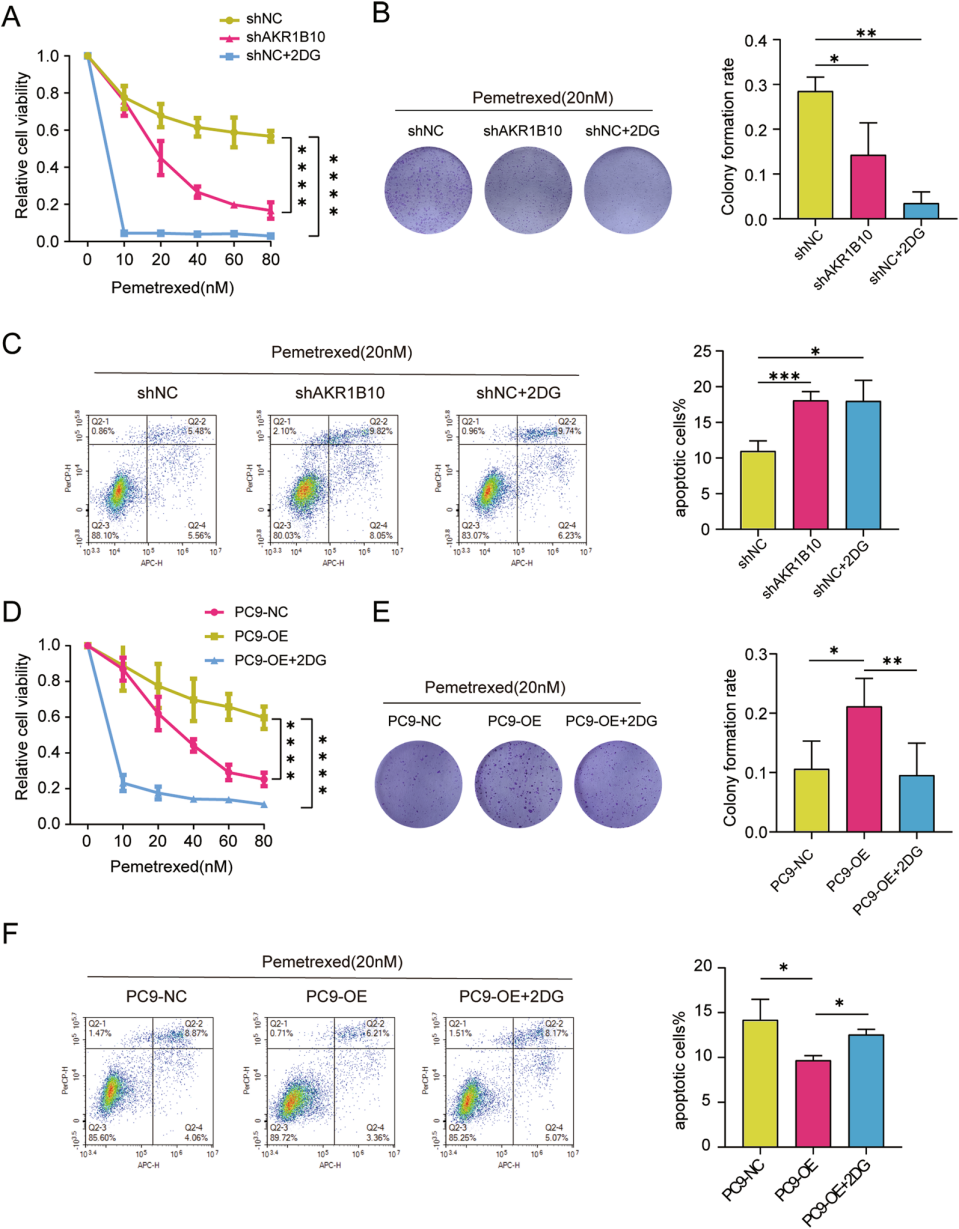
AKR1B10-mediated PEM resistance is dependent on the enhanced effects on anaerobic glycolysis. **A**, **D** Indicated cells were treated with different doses of PEM with or without 2-DG (2.5 mM) for 72 h and CCK-8 assays were performed to determine their viability. **B**, **E** Results of clonogenic assays showing cell survival of the indicated cells after treatment with PEM (20 nM) with or without 2-DG (2.5 mM). Pixel density quantification of clonogenic assays is shown as histogram. **C**, **F** Flow cytometry analysis of the indicated cells after treatment with PEM (20 nM) with or without 2-DG (2.5 mM) for 24 h. (n = 3, *p < 0.05, **p < 0.01, ****p < 0.0001, PC9-NC, PC9 transfected with negative control plasmid; PC9-OE, PC9 transfected with AKR1B10 plasmid. shNC, PC9-BrM3 cells transfected with negative control shRNA; shAKR1B10, PC9-BrM3 cells transfected with AKR1B10-targeted shRNA vector 1)

Warburg effect has been regarded as a hallmark of cancer that can promotes tumor initiation and drug resistance [26, 27]. We investigated whether the glycolysis is essential for the AKR1B10-mediated obtainment of PEM resistance in lung cancer BM cells. By using glycolysis inhibitors 2-Deoxy-d-glucose(2-DG) in combination with PEM, we found that the glycolytic inhibitor 2-DG reduced the PEM resistance of BrM3 cells without AKR1B10 silence (BrM3-shNC), as the same effect as AKR1B10 silence (Fig. 4A–C). Importantly, the increased drug resistance in PC9 cells brought by AKR1B10 overexpression (PC9-OE) was significantly abolished with 2-DG treatment (Fig. 4D–F), evidenced by cell viability assays, clonogenicity assays and cell apoptosis assays. Collectively, these results suggested that the Warburg effect is involved in the regulation of PEM resistance by AKR1B10.

#### RNA-seq identified that AKR1B10 facilitates the Warburg metabolism by regulating the expression of LDHA

**Fig. 5:**
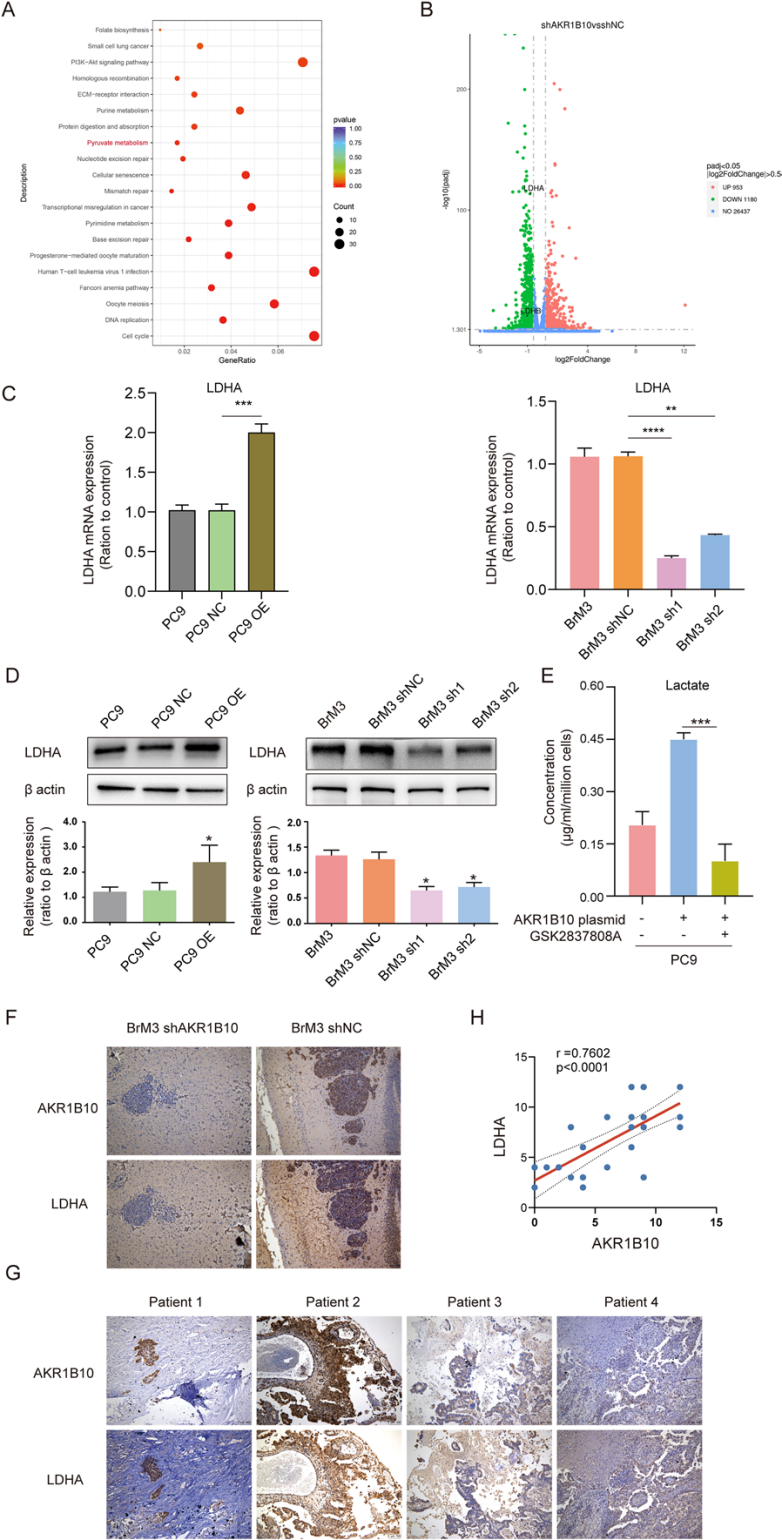
RNA-seq identified that AKR1B10 facilitates the Warburg metabolism by regulating the expression of glycolytic enzymes. **A** KEGG pathways enrichment analysis of downregulated differential genes. **B** Volcano plot shows the differential genes and the downregulated genes LDHA and LDHB were annotated. **C**, **D** The results of qPCR (**C**) and western blotting (**D**) indicating the mRNA and protein levels of LDHA in indicated cells, n = 3. **E** Cellular concentrations of lactate in indicted groups, n = 3. **F** IHC staining of AKR1B10 and LDHA in brain tumors isolated from mice after intracranial injection with BrM3-shNC or BrM3-shAKR1B10 cells. Scar bar, 100 µM. n = 3. **G** Representative images of AKR1B10 and LDHA staining in the metastasectomy surgical specimens from lung cancer BM patients. Scar bar, 100 µM. **H** Scatter diagram showing the correlation between the IRS scores of AKR1B10 and LDHA in surgical samples of 29 lung cancer patients with brain metastasis. (*p < 0.05, ***p < 0.001, ****p < 0.0001, PC9-NC, PC9 transfected with negative control plasmid; PC9-OE, PC9 transfected with AKR1B10 plasmid; shNC, PC9-BrM3 cells transfected with negative control shRNA; sh1, PC9-BrM3 cells transfected with AKR1B10-targeted shRNA vector 1; sh2, PC9-BrM3 cells transfected with AKR1B10-targeted shRNA vector 2; shAKR1B10, PC9-BrM3 cells transfected with AKR1B10-targeted shRNA vector 1)

To reveal the underlying mechanism that AKR1B10 facilitates the Warburg metabolism, we employed RNA-seq in PC9-BrM3 cells with or without AKR1B10 silence (BrM3-shNC/shAKR1B10). The Pearson correlation coefficient analysis indicated good reproducibility of RNA-seq data (Additional file 5: Fig. S2A). We used the absolute values of log_2_FoldChange > 0.5 and padj < 0.05 as the screening criteria to identify differential genes, and then mapped and enriched the downregulated differential genes in KEGG pathways. The pathway of pyruvate metabolism, which contains the glycolytic metabolism from pyruvate to lactate, was found enriched in BrM3-shAKR1B10 group compared with BrM3-shNC (Fig. 5A), and the exact differential genes enriched in the pyruvate metabolism were lactate dehydrogenases (LDHA, LDHB) (Fig. 5B), of which LDHA has a higher affinity for pyruvate, facilitating glycolytic process by preferentially converting pyruvate to lactate [28]. qPCR and western blotting verified that AKR1B10 silence leads to the downregulation of LDHA while overexpression of AKR1B10 caused the increased LDHA (Fig. 5C, D), revealing the mechanism on the regulation of AKR1B10 on the Warburg metabolism. Also, AKR1B10 displayed a similar regulatory effect on LDHB (Additional file 5: Fig. S2C). To demonstrate that the AKR1B10-LDHA regulatory axis is the main pathway of AKR1B10-enhanced glycolysis, we further administered GSK2837808A, a potent and selective LDHA inhibitor, in PC9 cells overexpressing AKR1B10 (PC9-OE) and then assess the cellular lactate level, the results showed that GSK2837808A remarkably attenuated the increased lactate level caused by AKR1B10 overexpression in PC9 cells (Fig. 5E). Staining of brain tumors isolated from mice after intracranial injection also verified that the expression of LDHA was significantly decreased in BrM3-shAKR1B10 group, compared with BrM3-shNC group (Fig. 5F). We further collected the metastasectomy surgical specimens from lung cancer BM patients (n = 29) and assessed the correlation between AKR1B10 and LDHA expression by IHC staining, the data showed a significant positive correlation between AKR1B10 and LDHA (Fig. 5G, H), indicating the existence of AKR1B10-LDHA regulatory axis in lung cancer BM. According to the staining results, we categorised patients with AKR1B10 expression IRS score ≥ 6 as the high expression group (n = 20) while patients with AKR1B10 expression IRS score < 6 (n = 9) as the low expression group, and performed the survival analysis which showed that those patients with high AKR1B10 expression had a shorter overall survival (OS) (Additional file 5: Fig. S2D). Besides, phosphoinositide 3-kinase (PI3K) pathway, a key link modulates the multidrug resistance of cancers [29], was also enriched in the BrM3-shAKR1B10 group compared with BrM3-shNC (Fig. 5A, Additional file 5: Fig. S2B). Since a recent study notably defined the association between glycolysis and PI3K pathway that glycolysis constantly enhanced PI3K pathway to control T cell immunity [30], we confirmed the effects of glycolysis on PI3K in BrM3 cells by treating the cells with 2-DG. The results of western blotting showed that inhibition of glycolysis resulted in the significant suppression of PI3K pathway (Additional file 5: Fig. S2E). To summarize, the results suggested that AKR1B10 promotes the production of lactate by increasing the LDHA expression.

### Accumulated lactate boosts transcriptional activation of cell cycle-related gene ***CCNB1 ***by histone lactylation

**Fig. 6:**
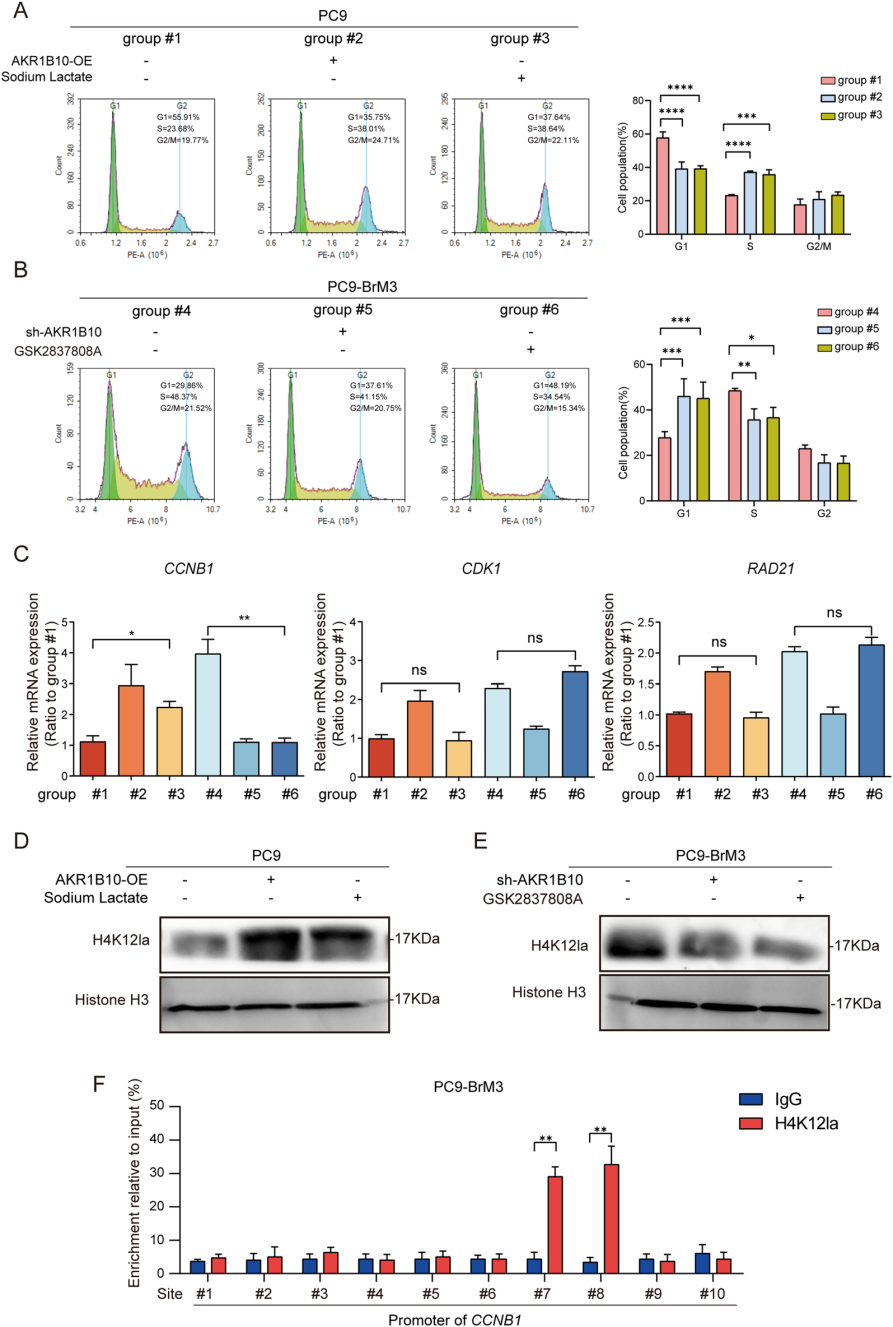
Accumulated lactate boosts transcriptional activation of cell cycle-related gene CCNB1 by histone lactylation. **A**, **B** Cell cycle assessed by flow cytometry assays in indicated cells. **C** The results of qPCR indicating the mRNA of *CCNB1, CDK1, RAD21* in indicated cells. **D**, **E** Histone of indicated cells were extracted and the level of H4K12la was detected by western blotting. The level of Histone H3 serves as control. **F** ChIP assay to detect histone lactylation level in *CCNB1* promoters in PC9-BrM3 cells. (n = 3, *p < 0.05, **p < 0.01, ***p < 0.001, ****p < 0.0001, group #1, PC9 transfected with negative control plasmid; group #2, PC9 transfected with AKR1B10 plasmid; group #3, PC9 transfected with negative control plasmid and treated with sodium lactate 50 mM for 24 h; group #4, PC9-BrM3 cells transfected with negative control shRNA; group #5, PC9-BrM3 cells transfected with AKR1B10-targeted shRNA vector; group #6, PC9-BrM3 cells transfected with negative control shRNA and treated with GSK2837808A 10µM for 24 h)

PEM is a cell cycle-specific antimetabolite drug which exerts antitumor effect mainly by interfering with the folic acid metabolic pathway during cell replication, and the results of RNA-seq showed that AKR1B10 knockdown exhibited the most significant effect on tumor cell cycle and DNA replication (Fig. 5A, Additional file 5: Fig. S2B); these suggested the potential mechanism that AKR1B10-increased lactate might promote chemoresistance in BM cells by regulating the cell cycle. Hence, we further explored the effects of LDHA-lactate on the cell cycle by examining and comparing the cell cycle progression of PC9 cells, PC9 cells with AKR1B10 overexpression or lactate exogenous supplementation (treatment with sodium lactate, 50mM for 24 h), BrM3 cells and BrM3 cells with AKR1B10 knockdown or LDHA inhibited (treatment with GSK2837808A, 10 µM for 24 h). The results of cell sorting analysis showed that both AKR1B10 overexpression and lactate supplementation in PC9 facilitated the cell cycle and DNA replication evidenced with the significant increased cell fraction in S stages (Fig. 6A). Consistently, silence of AKR1B10, as well as LDHA inhibition, caused significant cell cycle arrest in BM cells (Fig. 6B). These data indicated that AKR1B10-LDHA-lactate accelerates DNA replication and cell cycle in BM cells, suggesting the mechanism by which AKR1B10 mediates the PEM acquired drug-resistance in BM cells.

To ﻿further determine the regulation mechanism of lactate on cell cycle, we assessed the transcriptional level of *CCNB1, CDK1, RAD21* (three top cell-cycle related genes that were downregulated along with AKR1B10 silence indicated by RNA-seq). The results of qPCR showed that among these three AKR1B10-regulating genes, only the transcription of *CCNB1* was regulated by lactate levels (either lactate supplementation or LDHA inhibition, Fig. 6C), indicating that AKR1B10 derived lactate regulates cell cycle by activating transcription of *CCNB1.* Given the latest studies reporting lactate as a modulator of gene transcription through histone lactylation [31], we assessed the changes in lactylation in PC9 cells and BM cells. Western blot analysis showed that the global lactylation levels in BM cells, as well as PC9 cells treated with exogenous sodium lactate, were dramatically elevated compared with parental PC9 cells, and a predominant band appeared near 15 kDa, which may represent histone (Additional file 5: Fig. S3). Several common histone lactylation sites were further detected, and the results showed that, as similar as the change trends of cell cycle and transcription of *CCNB1*, the lactylation level of histone H4K12 (H4K12la) was increased in PC9 cells with AKR1B10 overexpression or lactate exogenous supplementation compared with PC9 cells (Fig. 6D), or decreased in BrM3 cells with AKR1B10 knockdown or LDHA inhibition compared with BrM3 cells (Fig. 6E). Subsequent ChIP assay using anti-H4K12la antibody was carried out to confirm that AKR1B10 induced histone lactylation in *CCNB1* promoter in BM cells (Fig. 6F). Taken together, these data revealed the mechanism that enriched lactate by AKR1B10 promotes transcriptional activation of the cell cycle-related gene *CCNB1* by histone lactylation.

## Discussion

BM is the leading cause of poor prognosis in NSCLC patients. Pemetrexed is one of the preferred agents for non-squamous NSCLC with BM [32, 33]; however, it displays limited therapeutic efficacy against BM due to drug resistance and the blood–brain barrier (BBB). Recent studies have shown that impaired BBB allows tumor cells to invade the brain parenchyma and form brain metastases [15, 34], which also means the barrier that prevents the entry of drugs is broken, supporting the possibility that intrinsic reprogramming of metastatic cells takes an essential part in the development of chemoresistance. In the present work, we used the previously constructed highly brain metastatic lung cancer cells (PC9-BrM3) and confirmed the acquired resistance of brain metastatic cells to PEM compared to it parental PC9 cells, which gives a reasonable explanation for the poor clinical response to chemotherapy in BM populations. Hence, it’s necessary to explore the intrinsic factors causing the altered drug sensitivity of BM.

AKR1B10 is a member of the AR superfamily and has been identified as a novel protein in human hepatocellular carcinoma (HCC) [35]. In our recent study, we demonstrated for the first time that AKR1B10, a protein that has been reported to be associated with drug resistance in a variety of primary tumors, is significantly elevated in PC9-BrM3 cells and NSCLC BM patients [15]. Here we found that suppression of AKR1B10 significantly attenuated the acquired PEM resistance in PC9-BrM3 cells, suggesting that AKR1B10 plays an important role in mediating the PEM resistance in BM.

Metabolic reprogramming is a hallmark of malignancy. Recent work has demonstrated the complexity of metabolic reprogramming in tumor metastasis, where the metabolic properties and preferences of tumors alter during cancer progression, which produces different tumor characteristics, such as acquisition of drug resistance, between primary and metastatic cancers [36]. Our previous research employed metabolomics in lung cancer BM subpopulations (PC9-BrMs) and its parental PC9, revealing a greatly altered metabolic profile of BM cells [17]. In the course of these recognitions, we propose that metabolic reprogramming contributes to acquired drug resistance in BM. By profiling the metabolism alternations brought by AKR1B10, we confirmed that AKR1B10 promotes the PEM resistance by facilitating the Warburg effect (aerobic glycolysis). It is worth mentioning that the effect of AKR1B10 on tumor metabolic reprogramming displays significant heterogeneity. In breast cancer, AKR1B10^High^ tumor cells, which are associated with an increased incidence of lung metastatic relapse, are characterized with reduced glycolytic capacity and dependency on glucose as fuel source but increased utilisation of fatty acid oxidation (FAO) [37]. Similarly, a recent study claimed that Fidarestat induces glycolysis of NK cells through decreasing AKR1B10 expression to inhibit hepatocellular carcinoma progression, especially lung metastasis [38]. Here we identified an opposing role of AKR1B10 in lung cancer derived brain metastatic cells that AKR1B10 presents a facilitative effect on anaerobic glycolysis. We speculate that there are differences in AKR1B10 related regulatory metabolic mechanisms in different tumors and cell types, as AKR1B10 has long been regarded to exhibit distinct effects in a variety of tumors [11, 39–42]. For lung and liver, which are characterized by a high level of oxidative stress and create a challenging microenvironment for metastatic tumor cells due to the high concentrations of oxygen and exposure to toxic compounds, increased AKR1B10 activity could serve to protect tumor cells from oxidative stress-induced damage which impairs the FAO metabolism [43, 44], leading to the a rebalancing of cellular metabolism in glycolysis and fatty acids. Brain microenvironment, which is characterized by presence of BBB and high oxygen and glucose demands, shows a high level of specificity compared with other organs. It is reasonable that tumor cells adapt different metabolic reprogramming in the face of different metastatic microenvironments. The metabolic regulatory heterogeneity of AKR1B10 for different tumors and in the face of specific metastatic microenvironments deserves to be further explored in future studies.

The Warburg effect is considered a metabolic hallmark in malignant tumors. It refers to the metabolic shift that tumor cells preferentially convert glucose to pyruvate (followed by lactate formation) rather than oxidative phosphorylation to meet the energy requirements under physiological oxygen conditions. It is characterized the accumulation of lactate which is the end product of glycolysis. The accumulated lactate is thought to drive tumor development, on the one hand, lactate leads to tumor acidosis which synergistically promotes tumor progress and resistance to antineoplastic drugs [45], on the other hand, the non-metabolic functions of lactate have been highlighted recently that lactate-derived lactylation of protein lysine residues (Kla) serves as an epigenetic modification to facilitate tumor malignant behaviors [31, 46, 47]. Lactylation, a post-translational modification originally reported in 2019 [31], has recently received increased attention in various disease researches. Up to now, studies on Kla have concentrated on both histone and non-histone proteins [48]. Histone Kla has been shown to facilitate the development of tumors. For example, Yu et al. reported that histone Kla drives tumorigenesis by promoting the expression of the m(6)A reading protein YTHDF2 in ocular melanoma [46]; Yang J et al. found that histone Kla promotes the progression of clear cell renal cell carcinoma through activation of platelet-derived growth factor receptor beta (PDGFRβ) transcription [49]; Jiang et al. confirmed that histone Kla regulates the expressions of a series of glycolysis-related enzymes and modulates cellular metabolism in non-small cell lung cancer [50]. Here we demonstrated that the enriched lactate promotes transcriptional activation of cell cycle-related gene *CCNB1* by histone lactylation (H4K12la) in lung cancer brain metastatic cells, supporting that histone lactylation also plays an important role in the acquirement of chemoresistance.

The level of LDHA, which is the key enzyme converting pyruvate to lactate, reflects the ability of malignant cells to metabolize pyruvate to lactate. Here we identified that AKR1B10 regulates the transcription of LDHA, clarifying the mechanism that AKR1B10 enhances the glycolysis metabolism in BM cells. Besides, we found that AKR1B10 also has an impact on the expression of LDHB. LDHA and LDHB are shown to jointly be involved in mediating the bidirectional conversion of pyruvate and lactate. LDHA has a higher affinity for pyruvate, preferentially converting pyruvate to lactate whereas LDHB possess a higher affinity for lactate, preferentially converting lactate to pyruvate [51]. Since this work determined that high levels of AKR1B10 promote the expression of both LDHA and LDHB, it could be explained that in the case of LDHA activation by AKR1B10, the substrate pyruvate remained in a state of significant accumulation accompanied with the high expression of AKR1B10, indicating a hyperactive state in the bidirectional conversion of pyruvate and lactate.

## Conclusions

**Fig. 7:**
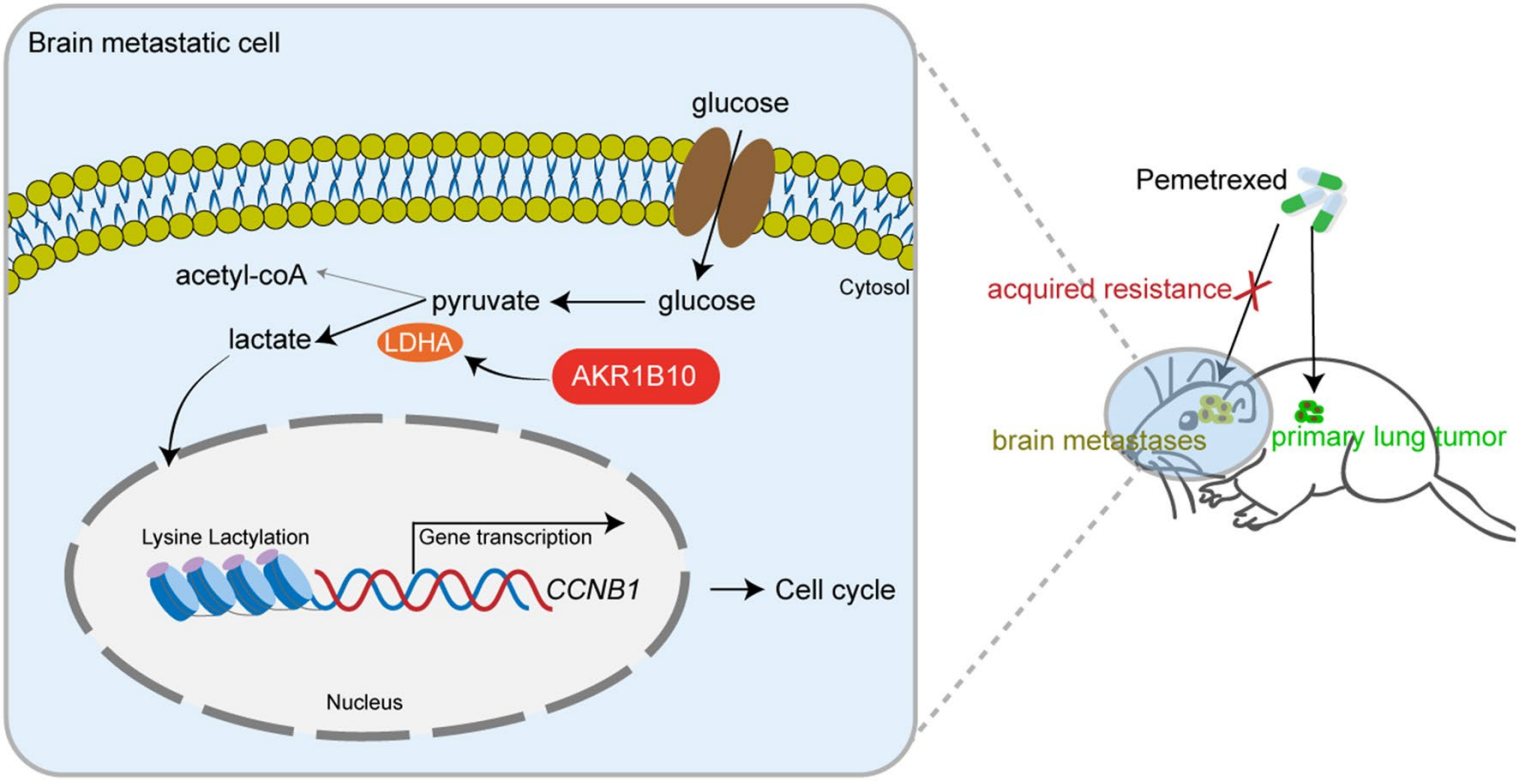
Illustration of the potential mechanisms by which AKR1B10 induces PEM chemotherapeutic resistance in lung cancer brain metastasis. In brain metastatic lung cancer cells, elevated AKR1B10 promotes glycolysis through upregulating the expression of LDHA and the resulting increased lactate induces transcriptional activation of the cell cycle-related gene *CCNB1* by histone lactylation, leading to the chemotherapeutic resistance to PEM.

In summary, the present study reveals that elevated AKR1B10 enhances glycolysis through regulation of LDHA and the resulting elevated lactate promotes transcriptional activation of the cell cycle-related gene *CCNB1* by histone lactylation, inhibiting the susceptibility of lung cancer BM cells to PEM (Fig. 7). These findings outline the importance of AKR1B10 in the BM resistance to tumor therapy via a way of metabolic reprogramming and epigenetic modification, providing new strategies to overcome chemotherapy resistance in lung cancer BM.

### Supplementary information

**Additional file 1: Table S1.** The primers used for qPCR analysis.

**Additional file 2: Table S2.** The codes reference resource for R packages.

**Additional file 3: Table S3.** Differential metabolites identified by metabolomics.

**Additional file 4: Table S4.** The quantitative source data.

**Additional file 5: Fig. S1.** Metabolic profiling analysis in indicated cells. (A) A typical total ion chromatogram of metabolic profiling. (B) The RSD distribution of metabolic features in QC samples. **Fig. S2.** RNA-seq analysis in indicated cells. (A) The Pearson correlation coefficient analysis of RNA-seq data. (B) KEGG pathway enrichment analysis of RNA-seq. The numbers on the bars represent the differential genes involved. (C) The results of qPCR indicating the mRNA levels of LDHB in indicated cells. (D) Survival analysis of lung cancer BM patients with high (n=20) or low (n=9) AKR1B10 expression. (E) The results of western blotting indicating the protein levels of PI3K pathway in BrM3 cells, with or without AKR1B10 knockdown, or with 2-DG (2.5mM) treatment. p-PI3K, phosphorylated; T-PI3K, total PI3K; p-AKT, phosphorylated AKT; T-AKT, total AKT. (n=3, *p<0.05, **p<0.01, PC9-NC, PC9 transfected with negative control plasmid; PC9-OE, PC9 transfected with AKR1B10 plasmid; shNC, PC9-BrM3 cells transfected with negative control shRNA; sh1, PC9-BrM3 cells transfected with AKR1B10-targeted shRNA vector 1; sh2, PC9-BrM3 cells transfected with AKR1B10-targeted shRNA vector 2; shAKR1B10, PC9-BrM3 cells transfected with AKR1B10-targeted shRNA vector 1). **Fig. S3.** Accumulated lactate increased the global lactylation levels. Western blot analysis showed that the global lactylation levels in parental PC9 cells, BrM cells, as well as PC9 cells treated with exogenous sodium lactate (Nala) for 24h.

## Data Availability

The mass spectrometry data have been deposited to the ProteomeXchange Consortium (http://proteomecentral.proteomexchange.org) via the iProX partner repository with the dataset identifier PXD043632 and the RNA sequencing raw data has been deposited in NCBI GEO with accession number GSE211215. Furthermore, the quantitative source data from other assays has been provided in the Additional file 4: Table S4.

## References

[1] Achrol AS, Rennert RC, Anders C (2019). Brain metastases. Nat Rev Dis Primers.

[2] Steeg PS, Camphausen KA, Smith QR (2011). Brain metastases as preventive and therapeutic targets. Nat Rev Cancer.

[3] Shi Y, Sun Y, Yu J (2017). [China experts Consensus on the diagnosis and treatment of Brain Metastases of Lung Cancer (2017 version)]. Zhongguo Fei Ai Za Zhi.

[4] Gadgeel S, Rodríguez-Abreu D, Speranza G (2020). Updated analysis from KEYNOTE-189: Pembrolizumab or Placebo Plus Pemetrexed and Platinum for previously untreated metastatic nonsquamous non-small-cell Lung Cancer. J Clin Oncol.

[5] Schiller JH, Harrington D, Belani CP (2002). Comparison of four chemotherapy regimens for advanced non-small-cell lung cancer. N Engl J Med.

[6] Bailon O, Chouahnia K, Augier A (2012). Upfront association of carboplatin plus pemetrexed in patients with brain metastases of lung adenocarcinoma. Neuro Oncol.

[7] Barlesi F, Gervais R, Lena H (2011). Pemetrexed and cisplatin as first-line chemotherapy for advanced non-small-cell lung cancer (NSCLC) with asymptomatic inoperable brain metastases: a multicenter phase II trial (GFPC 07 – 01). Ann Oncol.

[8] Luo DX, Huang MC, Ma J, Gao Z, Liao DF, Cao D (2011). Aldo-Keto reductase family 1, member B10 is secreted through a lysosome-mediated non-classical pathway. Biochem J.

[9] Banerjee S (2021). Aldo keto reductases AKR1B1 and AKR1B10 in cancer: molecular mechanisms and signaling networks. Adv Exp Med Biol.

[10] Cheng BY, Lau EY, Leung HW (2018). IRAK1 augments cancer stemness and drug resistance via the AP-1/AKR1B10 signaling cascade in hepatocellular carcinoma. Cancer Res.

[11] Zhang T, Guan G, Zhang J (2022). E2F1-mediated AUF1 upregulation promotes HCC development and enhances drug resistance via stabilization of AKR1B10. Cancer Sci.

[12] Zhong L, Shen H, Huang C, Jing H, Cao D (2011). AKR1B10 induces cell resistance to daunorubicin and idarubicin by reducing C13 ketonic group. Toxicol Appl Pharmacol.

[13] Matsunaga T, Kawabata S, Yanagihara Y (2019). Pathophysiological roles of autophagy and aldo-keto reductases in development of doxorubicin resistance in gastrointestinal cancer cells. Chem Biol Interact.

[14] Endo S, Xia S, Suyama M (2017). Synthesis of potent and selective inhibitors of Aldo-Keto reductase 1B10 and their efficacy against proliferation, metastasis, and cisplatin resistance of lung cancer cells. J Med Chem.

[15] Liu W, Song J, Du X (2019). AKR1B10 (Aldo-keto reductase family 1 B10) promotes brain metastasis of lung cancer cells in a multi-organ microfluidic chip model. Acta Biomater.

[16] Geisler JA, Spehar JM, Steck SA (2020). Modeling brain metastases through intracranial injection and magnetic resonance imaging. J Vis Exp.

[17] Liu W, Zhou Y, Duan W (2021). Glutathione peroxidase 4-dependent glutathione high-consumption drives acquired platinum chemoresistance in lung cancer-derived brain metastasis. Clin Transl Med.

[18] Zhou Y, Song R, Zhang Z (2016). The development of plasma pseudotargeted GC-MS metabolic profiling and its application in bladder cancer. Anal Bioanal Chem.

[19] Bernstein A, Reichert S, Südkamp NP (2020). Expression of xylosyltransferases I and II and their role in the pathogenesis of arthrofibrosis. J Orthop Surg Res.

[20] Storey JD (2002). A direct approach to false discovery rates. J R Statist Soc B.

[21] Chong J, Soufan O, Li C (2018). MetaboAnalyst 4.0: towards more transparent and integrative metabolomics analysis. Nucleic Acids Res.

[22] Love MI, Huber W, Anders S (2014). Moderated estimation of fold change and dispersion for RNA-seq data with DESeq2. Genome Biol.

[23] Kong D, Mao JH, Li H (2022). Effects and associated transcriptomic landscape changes of methamphetamine on immune cells. BMC Med Genomics.

[24] Hochrein SM, Wu H, Eckstein M (2022). The glucose transporter GLUT3 controls T helper 17 cell responses through glycolytic-epigenetic reprogramming. Cell Metab.

[25] Martínez-Reyes I, Chandel NS (2021). Cancer metabolism: looking forward. Nat Rev Cancer.

[26] Ma L, Liu W, Xu A (2020). Activator of thyroid and retinoid receptor increases sorafenib resistance in hepatocellular carcinoma by facilitating the Warburg effect. Cancer Sci.

[27] Bhattacharya B, Mohd Omar MF, Soong R (2016). The Warburg effect and drug resistance. Br J Pharmacol.

[28] Huo N, Cong R, Sun ZJ (2021). STAT3/LINC00671 axis regulates papillary thyroid tumor growth and metastasis via LDHA-mediated glycolysis. Cell Death Dis.

[29] Liu R, Chen Y, Liu G (2020). PI3K/AKT pathway as a key link modulates the multidrug resistance of cancers. Cell Death Dis.

[30] Xu K, Yin N, Peng M (2021). Glycolysis fuels phosphoinositide 3-kinase signaling to bolster T cell immunity. Science.

[31] Zhang D, Tang Z, Huang H (2019). Metabolic regulation of gene expression by histone lactylation. Nature.

[32] He G, Xiao X, Zou M, Zhang C, Xia S (2016). Pemetrexed/cisplatin as first-line chemotherapy for advanced lung cancer with brain metastases: a case report and literature review. Medicine (Baltim).

[33] Bearz A, Garassino I, Tiseo M (2010). Activity of pemetrexed on brain metastases from non-small cell lung cancer. Lung Cancer.

[34] Li B, Wang C, Zhang Y (2013). Elevated PLGF contributes to small-cell lung cancer brain metastasis. Oncogene.

[35] Ye X, Li C, Zu X (2019). A large-scale multicenter study validates Aldo-Keto reductase family 1 member B10 as a prevalent serum marker for detection of hepatocellular carcinoma. Hepatology.

[36] Faubert B, Solmonson A, DeBerardinis RJ (2020). Metabolic reprogramming and cancer progression. Science.

[37] van Weverwijk A, Koundouros N, Iravani M (2019). Metabolic adaptability in metastatic breast cancer by AKR1B10-dependent balancing of glycolysis and fatty acid oxidation. Nat Commun.

[38] Wu T, Ke Y, Tang H, Liao C, Li J, Wang L (2021). Fidarestat induces glycolysis of NK cells through decreasing AKR1B10 expression to inhibit hepatocellular carcinoma. Mol Ther Oncolytics.

[39] Taskoparan B, Seza EG, Demirkol S (2017). Opposing roles of the aldo-keto reductases AKR1B1 and AKR1B10 in colorectal cancer. Cell Oncol (Dordr).

[40] Yao Y, Wang X, Zhou D (2020). Loss of AKR1B10 promotes colorectal cancer cells proliferation and migration via regulating FGF1-dependent pathway. Aging.

[41] Shao X, Wu J, Yu S, Zhou Y, Zhou C (2021). AKR1B10 inhibits the proliferation and migration of gastric cancer via regulating epithelial-mesenchymal transition. Aging.

[42] Endo S, Matsunaga T, Nishinaka T (2021). The role of AKR1B10 in physiology and pathophysiology. Metabolites.

[43] Schafer ZT, Grassian AR, Song L (2009). Antioxidant and oncogene rescue of metabolic defects caused by loss of matrix attachment. Nature.

[44] Carracedo A, Cantley LC, Pandolfi PP (2013). Cancer metabolism: fatty acid oxidation in the limelight. Nat Rev Cancer.

[45] Vaupel P, Schmidberger H, Mayer A (2019). The Warburg effect: essential part of metabolic reprogramming and central contributor to cancer progression. Int J Radiat Biol.

[46] Yu J, Chai P, Xie M (2021). Histone lactylation drives oncogenesis by facilitating m(6)a reader protein YTHDF2 expression in ocular melanoma. Genome Biol.

[47] Gu J, Zhou J, Chen Q (2022). Tumor metabolite lactate promotes tumorigenesis by modulating MOESIN lactylation and enhancing TGF-β signaling in regulatory T cells. Cell Rep.

[48] Li X, Yang Y, Zhang B (2022). Lactate metabolism in human health and disease. Signal Transduct Target Ther.

[49] Yang J, Luo L, Zhao C (2022). A positive feedback loop between inactive VHL-triggered histone lactylation and PDGFRβ signaling drives clear cell renal cell carcinoma progression. Int J Biol Sci.

[50] Jiang J, Huang D, Jiang Y (2021). Lactate modulates cellular metabolism through histone lactylation-mediated gene expression in non-small cell lung cancer. Front Oncol.

[51] Urbańska K, Orzechowski A (2019). Unappreciated role of LDHA and LDHB to control apoptosis and autophagy in Tumor cells. Int J Mol Sci.

