# Supplementary material for: Warburg effect enhanced by AKR1B10 promotes acquired resistance to pemetrexed in lung cancer-derived brain metastasis
Source: J Transl Med. 2023 Aug 16;21:547. doi: 10.1186/s12967-023-04403-0 (PMC10428599; doi:10.1186/s12967-023-04403-0)
Supplement: Supplementary file 1 — Additional file 1: Table S1. The primers used for qPCR analysis. [file 12967_2023_4403_MOESM1_ESM.docx]

**Table S1**

| **Site** | **Primer** | **Product length** |
| --- | --- | --- |
| #1 | F: 5' AGGCAGGAGAATGGAGTGA 3'  R: 5' CGAGAGGCAAGGGGCGAAG 3' | 173bp |
| #2 | F: 5' GAGTGAGAATGGATGTTGA 3'  R: 5' TTACCCTTGCTCTCCTCTG 3' | 117bp |
| #3 | F: 5' GCTCTTATACCGTTGCTCT 3'  R: 5' TTTTTGCCTTCTACTCCTC 3' | 101bp |
| #4 | F: 5' TTTCCCATGAGAGGCAAATACATGT 3'  R: 5' ATACTTAGACCAGTTGCCA 3' | 113bp |
| #5 | F: 5' AACAACACGGATACACCCC 3'  R: 5' CTGAAGCTCAGCGACTTAG 3' | 111bp |
| #6 | F: 5' CAACGATCAAATGAAAGAA 3'  R: 5' TTGGAAGGTGACAGAGGCG 3' | 145bp |
| #7 | F: 5' ACTGGCTTCACTGCTCTCC 3'  R: 5' TCCCTTCCCATTGGCGGCT 3' | 187bp |
| #8 | F: 5' CCAATGGGAAGGGAGTGAG 3'  R: 5' AGCGGGGAGAAGCAGAACA 3' | 173bp |
| #9 | F: 5' CTGCTGCCTGCTCTCCCTG 3'  R: 5' GCTCTCTTGGTGAGTCCCC 3' | 103bp |
| #10 | F: 5' AATGTTTTGGGGAGGTGGA 3'  R: 5' CTCTCTGGGAAGGGGGTTA 3' | 103bp |
