# Supplementary material for: Warburg effect enhanced by AKR1B10 promotes acquired resistance to pemetrexed in lung cancer-derived brain metastasis
Source: J Transl Med. 2023 Aug 16;21:547. doi: 10.1186/s12967-023-04403-0 (PMC10428599; doi:10.1186/s12967-023-04403-0)
Supplement: Supplementary file 5 — Additional file 5: Fig. S1. Metabolic profiling analysis in indicated cells. (A) A typical total ion chromatogram of metabolic profiling. (B) The RSD distribution of metabolic features in QC samples. Fig. S2. RNA-seq analysis in indicated cells. (A) The Pearson correlation coefficient analysis of RNA-seq data. (B) KEGG pathway enrichment analysis of RNA-seq. The numbers on the bars represent the differential genes involved. (C) The results of qPCR indicating the mRNA levels of LDHB in indicated cells. (D) Survival analysis of lung cancer BM patients with high (n=20) or low (n=9) AKR1B10 expression. (E) The results of western blotting indicating the protein levels of PI3K pathway in BrM3 cells, with or without AKR1B10 knockdown, or with 2-DG (2.5mM) treatment. p-PI3K, phosphorylated; T-PI3K, total PI3K; p-AKT, phosphorylated AKT; T-AKT, total AKT. (n=3, *p<0.05, **p<0.01, PC9-NC, PC9 transfected with negative control plasmid; PC9-OE, PC9 transfected with AKR1B10 plasmid; shNC, PC9-BrM3 cells transfected with negative control shRNA; sh1, PC9-BrM3 cells transfected with AKR1B10-targeted shRNA vector 1; sh2, PC9-BrM3 cells transfected with AKR1B10-targeted shRNA vector 2; shAKR1B10, PC9-BrM3 cells transfected with AKR1B10-targeted shRNA vector 1). Fig. S3. Accumulated lactate increased the global lactylation levels. Western blot analysis showed that the global lactylation levels in parental PC9 cells, BrM cells, as well as PC9 cells treated with exogenous sodium lactate (Nala) for 24h. [file 12967_2023_4403_MOESM5_ESM.docx]

**Materials and methods**

1. **RNA-sequencing analysis**

1.1 RNA quantification and qualification

RNA integrity was assessed using the RNA Nano 6000 Assay Kit of the Bioanalyzer

2100 system (Agilent Technologies, CA, USA).
1.2 Library preparation for Transcriptome sequencing

Total RNA was used as input material for the RNA sample preparations. Briefly, mRNA was purified from total RNA using poly-T oligo-attached magnetic beads. Fragmentation was carried out using divalent cations under elevated temperature in First Strand Synthesis Reaction Buffer(5X). First strand cDNA was synthesized using random hexamer primer and M-MuLV Reverse Transcriptase, then use RNaseH to degrade the RNA. Second strand cDNA synthesis was subsequently performed using DNA Polymerase I and dNTP. Remaining overhangs were converted into blunt ends via exonuclease/polymerase activities. After adenylation of 3’ ends of DNA fragments, Adaptor with hairpin loop structure were ligated to prepare for hybridization. In order to select cDNA fragments of preferentially 370~420 bp in length, the library fragments were purified with AMPure XP system (Beckman Coulter, Beverly, USA). Then PCR was performed with Phusion High-Fidelity DNA

polymerase, Universal PCR primers and Index (X) Primer. At last, PCR products were purified

by AMPure XP system and library quality was assessed on the Agilent Bioanalyzer 2100 system.

1.3 Clustering and sequencing

The clustering of the index-coded samples was performed on a cBot Cluster Generation System using TruSeq PE Cluster Kit v3-cBot-HS (Illumia) according to the manufacturer’s instructions. After cluster generation, the library preparations were sequenced on an Illumina Novaseq platform and 150 bp paired-end reads were generated.

- 1. Data Analysis

Raw data (raw reads) of fastq format were firstly processed through in-house perl scripts. In this step, clean data (clean reads) were obtained by removing reads containing adapter, reads containing N base and low quality reads from raw data. At the same time, Q20, Q30 and GC content the clean data were calculated. All the downstream analyses were based on the clean data with high quality. Reference genome and gene model annotation files were downloaded from genome website directly. Index of the reference genome was built using Hisat2 v2.0.5 and paired-end clean reads were aligned to the reference genome using Hisat2 v2.0.5. FeatureCounts v1.5.0-p3 was used to count the reads numbers mapped to each gene. And then FPKM of each gene was calculated based on the length of the gene and reads count mapped to this gene. Differential expression analysis was performed using the DESeq2 R package (1.20.0). Genes with an adjusted P-value <0.05 found by DESeq2 were

assigned as differentially expressed. ClusterProfiler R package was used for Gene Ontology (GO) and KEGG enrichment analysis. The codes reference resource was provided in the Table S2.

**Figures**


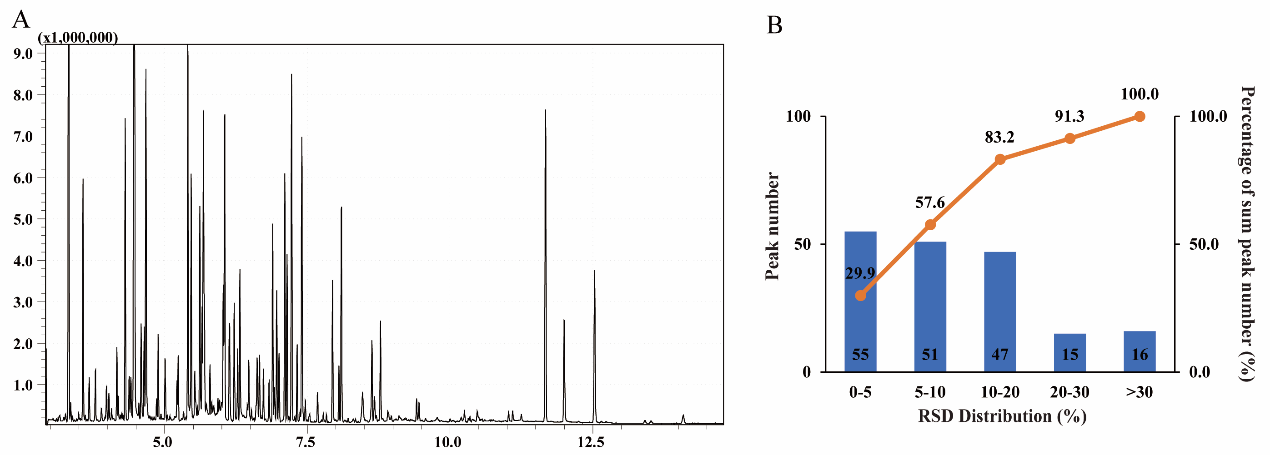


**Fig.S1** **Metabolic profiling analysis in indicated cells.** (A) A typical total ion chromatogram of metabolic profiling. (B) The RSD distribution of metabolic features in QC samples.


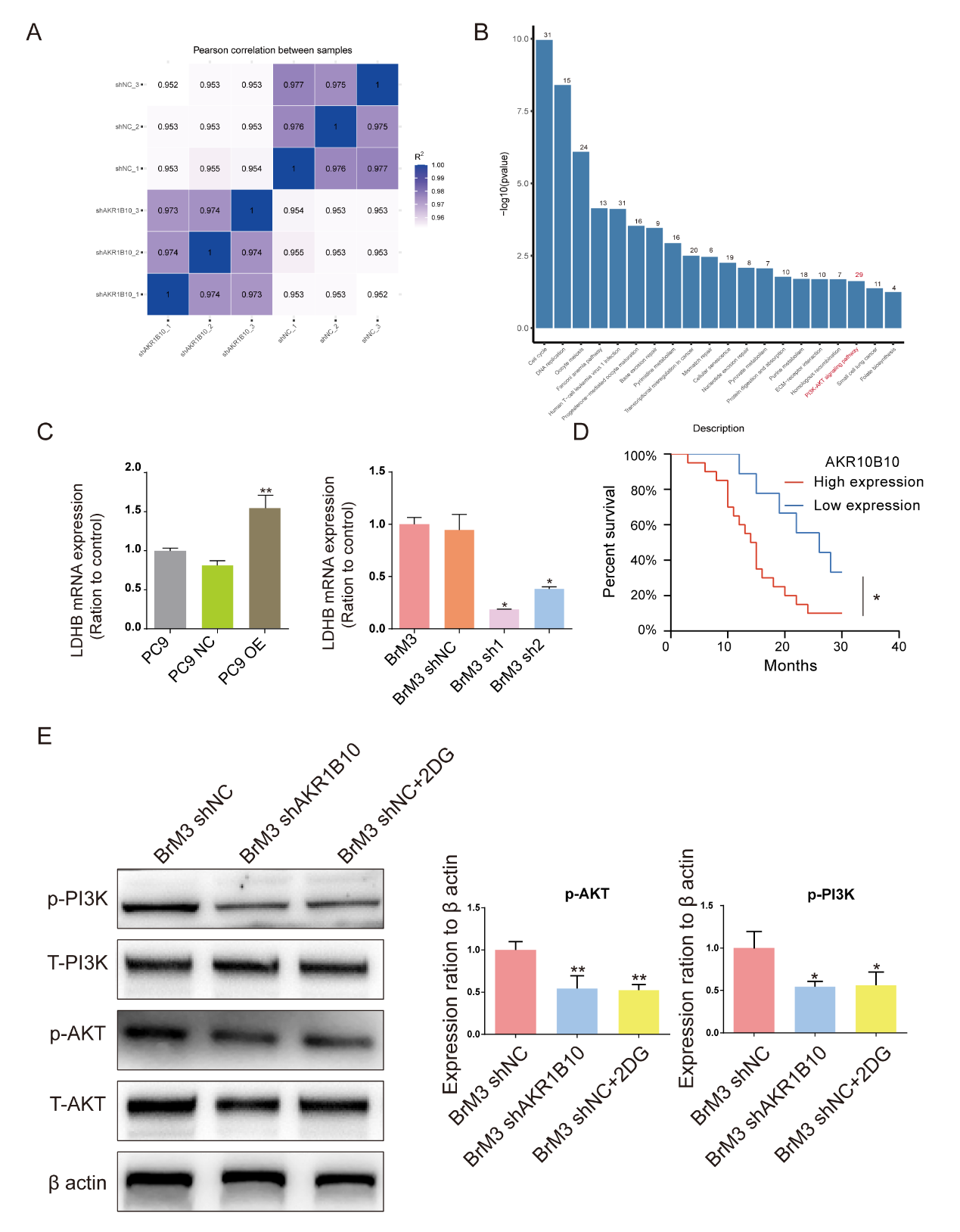


**Fig.S2** **RNA-seq analysis in indicated cells.** (A) The Pearson correlation coefficient analysis of RNA-seq data. (B) KEGG pathway enrichment analysis of RNA-seq. The numbers on the bars represent the differential genes involved. (C) The results of qPCR indicating the mRNA levels of LDHB in indicated cells. (D) Survival analysis of lung cancer BM patients with high (n=20) or low (n=9) AKR1B10 expression. (E) The results of western blotting indicating the protein levels of PI3K pathway in BrM3 cells, with or without AKR1B10 knockdown, or with 2-DG (2.5mM) treatment. p-PI3K, phosphorylated; T-PI3K, total PI3K; p-AKT, phosphorylated AKT; T-AKT, total AKT. (n=3, *p<0.05, **p<0.01, PC9-NC, PC9 transfected with negative control plasmid; PC9-OE, PC9 transfected with AKR1B10 plasmid; shNC, PC9-BrM3 cells transfected with negative control shRNA; sh1, PC9-BrM3 cells transfected with AKR1B10-targeted shRNA vector 1; sh2, PC9-BrM3 cells transfected with AKR1B10-targeted shRNA vector 2; shAKR1B10, PC9-BrM3 cells transfected with AKR1B10-targeted shRNA vector 1)


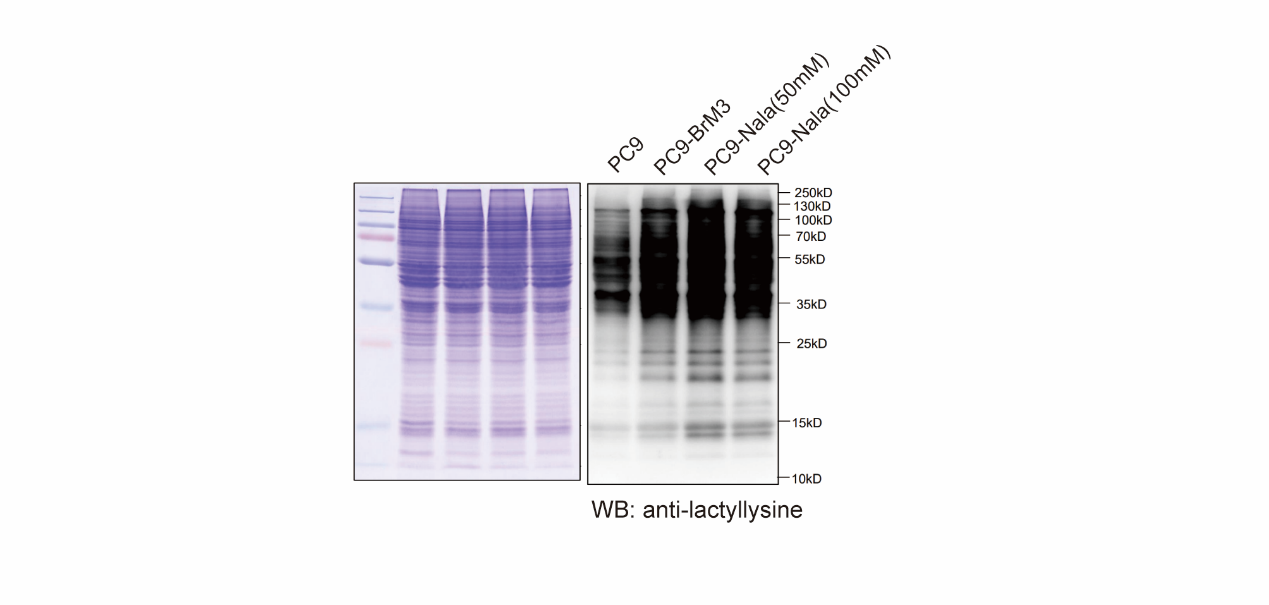


**Fig.S3 Accumulated lactate increased the global lactylation levels.** Western blot analysis showed that the global lactylation levels in parental PC9 cells, BrM cells, as well as PC9 cells treated with exogenous sodium lactate (Nala) for 24h.
